# Establishment and validation of evaluation models for post-inflammatory pigmentation abnormalities

**DOI:** 10.3389/fimmu.2022.991594

**Published:** 2022-10-27

**Authors:** Yushan Zhang, Hongliang Zeng, Yibo Hu, Ling Jiang, Chuhan Fu, Lan Zhang, Fan Zhang, Xiaolin Zhang, Lu Zhu, Jinhua Huang, Jing Chen, Qinghai Zeng

**Affiliations:** ^1^ Department of Dermatology, The Third Xiangya Hospital, Central South University, Changsha, China; ^2^ Institute of Chinese Materia Medica, Hunan Academy of Chinese Medicine, Changsha, China

**Keywords:** post-inflammatory pigmentation abnormalities, melanogenesis, machine learning, evaluation models, IL-37

## Abstract

Post-inflammatory skin hyper- or hypo-pigmentation is a common occurrence with unclear etiology. There is currently no reliable method to predict skin pigmentation outcomes after inflammation. In this study, we analyzed the 5 GEO datasets to screen for inflammatory**-**related genes involved in melanogenesis, and used candidate cytokines to establish different machine learning (LASSO regression, logistic regression and Random Forest) models to predict the pigmentation outcomes of post-inflammatory skin. Further, to further validate those models, we evaluated the role of these candidate cytokines in pigment cells. We found that IL-37, CXCL13, CXCL1, CXCL2 and IL-19 showed high predictive value in predictive models. All models accurately classified skin samples with different melanogenesis-related gene scores in the training and testing sets (AUC>0.7). Meanwhile, we mainly evaluated the effects of IL-37 in pigment cells, and found that it increased the melanin content and expression of melanogenesis-related genes (MITF, TYR, TYRP1 and DCT), also enhanced tyrosinase activity. In addition, CXCL13, CXCL1, CXCL2 and IL-19 could down-regulate the expression of several melanogenesis-related genes. In conclusion, evaluation models basing on machine learning may be valuable in predicting outcomes of post-inflammatory pigmentation abnormalities. IL-37, CXCL1, CXCL2, CXCL13 and IL-19 are involved in regulating post-inflammatory pigmentation abnormalities.

## 1 Introduction

Inflammatory skin disorders are frequently associated with aberrant post-inflammatory pigmentation. UV radiation, burns, trauma and inflammation can cause skin hyper- or hypo-pigmentation (1), which affects appearance and take months or even years to subside (2). Various inflammatory skin diseases have different effects on skin pigmentation. For instance, psoriatic lesions are typically hypo-pigmented during active inflammation, while resolution of the inflammatory process increases the risk of hyper-pigmentation and to certain extent that of hypo-pigmentation as well (3, 4). In other words, the effects of inflammation on skin pigmentation are unpredictable.

Although the mechanisms underlying aberrant pigmentation remain elusive, a few inflammatory cytokines have been identified as potential factors. Pigmentation is the result of a well-coordinated process of melanogenesis, which involves melanin production and transport in the melanocytes, followed by its distribution and degradation in the keratinocytes (5, 6). Recent studies show that some cytokines can regulate melanogenesis. For example, IL-17 and TNF synergistically inhibit the PKA and MAPK pathways to reduce melanogenesis, whereas IL-1β, IL-4, IFN-γ, IL-6 and IL36-γ inhibit melanogenesis through blocking the NF-κB, JNK, JAK/STAT or other signaling pathways (7–11). In contrast, IL-33 and IL-18 enhance melanogenesis by activating the PKA and p38/MAPK signaling pathways (12–14). It is necessary to identify core inflammatory factors that regulate melanogenesis, and explore the underlying mechanisms in order to mitigate aberrant pigmentation following inflammation.

With the advent of transcriptome arrays, a considerable amount of transcriptomic data pertaining to different tissues, diseases etc. have been stored in public database, such as Gene Expression Omnibus (GEO). Analysis of these transcriptome datasets through machine learning methods (LASSO regression, logistic regression and Random Forest) can provide preliminary evidence for further studies. To this end, our aim was to analyze the available transcriptome data on skin inflammatory disorders in order to screen for inflammatory factors involved in the regulation of melanogenesis. In addition, this study also attempted to predict the pigmentation outcomes of post-inflammatory skin basing on machine learning methods.

## 2 Materials and methods

### 2.1 Bioinformatics analysis

#### 2.1.1 Datasets collection

**Figure 1:**
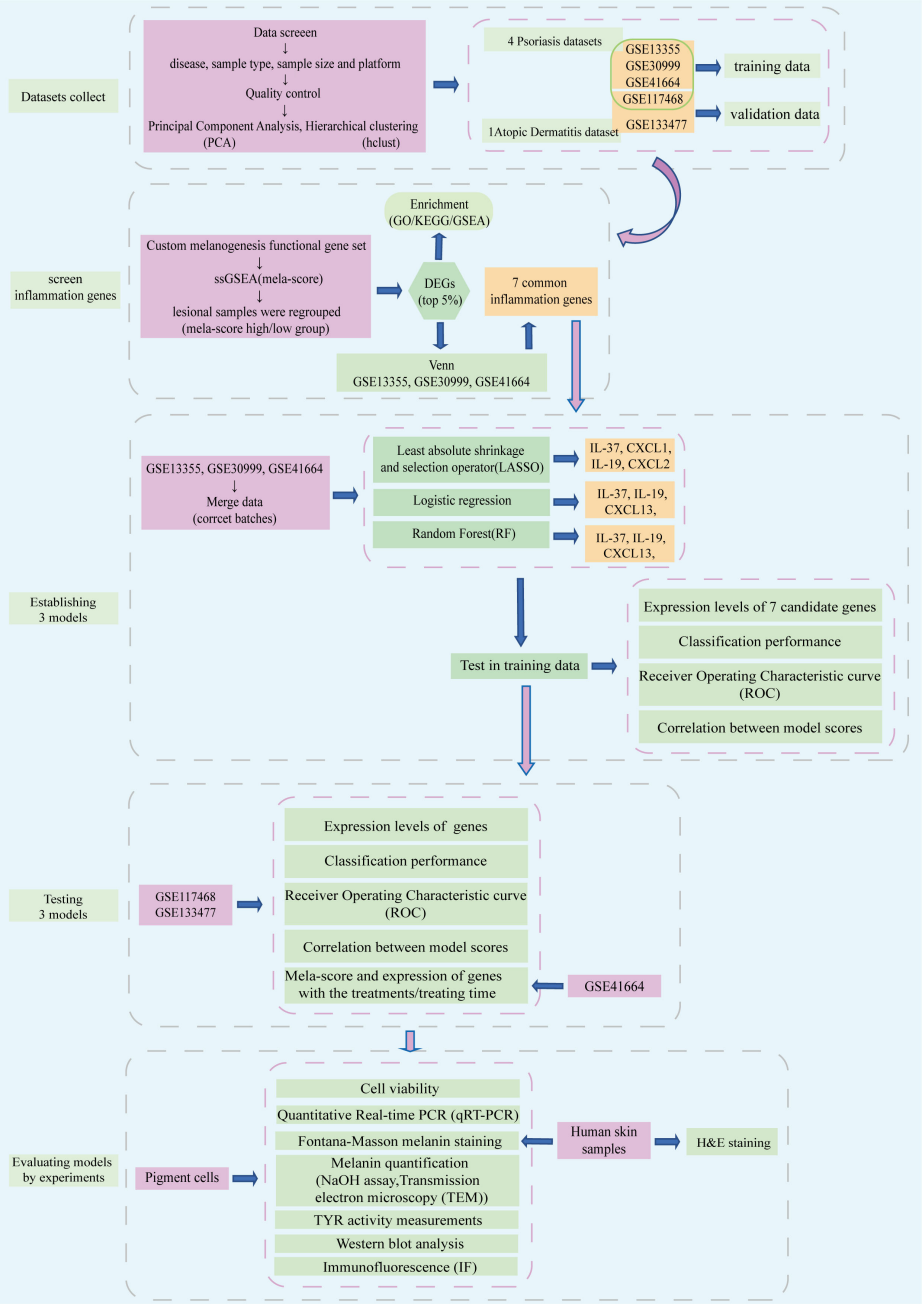
Flow chart of this study.

The flow chart of the research is shown in **Figure 1**. The public datasets used in this work were downloaded from GEO (http://www.ncbi.nlm.nih.gov/geo/). Datasets have been preliminary screened on the basis of disease (inflammatory skin disease), sample type (skin specimen), sample size (number of samples > 100) and platform (commonly used microarray platforms GPL570). Finally, three psoriasis datasets (GSE13355, GSE30999 and GSE41664) were selected as training data, one dataset each of atopic dermatitis (GSE133477) and psoriasis (GSE117468) were selected as the validation set (**Table 1**). Data was processed by R (version 4.0.3) on Linux system (Ubuntu, version 20.04). The quality of datasets was preliminary assessed based on gene expression levels (the expression data (log2 scaled, processed by R package limma) from GPL570 platform had good quality, no extremely low- or high-expressed probes/genes were found, thus no further screening was conducted), Principal Component Analysis (PCA) and Hierarchical clustering (hclust).

**Table 1:** Information of GEO datasets.

GEO accession	Platform	Size	Source tissue	Treatment
GSE13355 (15)	GPL570	180	Human skin	No treatment
GSE30999 (16)	GPL570	170	Human skin	No treatment
GSE41664 (17)	GPL570	157	Human skin	Etanercept
GSE117468 (18)	GPL570	565	Human skin	Brodalumanb
				Ustekinumab
GSE133477 (19)	GPL570	242	Human skin	Crisaborole

GPL570: [HG-U133_Plus_2] Affymetrix Human Genome U133 Plus 2.0 Array.

#### 2.1.2 Dataset processing and enrichment analysis

MITF, TYR, DCT, PMEL, MLANA, WNT4 and WNT2A were defined as a functional gene set of melanogenesis, and the weighted scores of the gene set were calculated by package gsva (version 1.38.2) through single-sample gene set enrichment analysis (ssGSEA). The lesion samples were regrouped according to the ssGSEA scores. Differentially expressed genes (DEGs) between the high- and low-score groups were identified using the limma package (version 3.46.0), and the significant DEGs were screened based on p-value (p < 0.05) and log_2_foldchange (absolute value in the top 5%). The DEGs were functionally annotated by Gene Ontology (GO) and Kyoto Encyclopedia of Genes and Genomes (KEGG) analyses, and the enriched functions were screened using package clusterProfiler (20) (version 4.3.0.991) and gene set enrichment analysis (GSEA).

#### 2.1.3 Establishment and validation of models

The lesion samples from the GSE13355, GSE30999 and GSE41664 datasets were pooled into the training set. The batches between datasets were corrected by package sva (version 3.38.0). Among different machine learning methods, Least absolute shrinkage and selection operator (LASSO, package glmnet) regression, logistic regression and Random Forest (RF, package randomForest), were commonly used in medical research (21) and chosen as representatives in our work. During model establishing, some parameters were set (LASSO: Lambda=0.461, RF: ntree=500, mtyr=0.2169). The performance of the models was evaluated by receiver operating characteristic (ROC) curve analysis on the testing datasets (GSE117468 and GSE133477). The data was visualized with R package ggplot2 and corrplot.

### 2.2 Experimental validation

#### 2.2.1 Reagents

Cytokines (IL-37, IL-19, CXCL1, CXCL2 and CXCL13) were purchased from Cloud-Clone Corp (Wuhan, China), and L-dopa and sodium deoxycholate from Solarbio (Beijing, China). TritonX-100 was purchased from Sigma-Aldrich. Cell Counting Kit-8 (CCK8) and 4% neutral paraformaldehyde were from Biosharp (Hefei, China). Fetal bovine serum (FBS) from Meisen (Zhejiang, China). Dulbecco’s modified Eagle medium (DMEM), penicillin/streptomycin/amphotericin B, penicillin-streptomycin (P/S), and non-essential amino acids (NEAA) were from Gibco (Maryland, USA). Primary antibodies specific for GAPDH (#AP0066, Bioworld), TYR (BS1484, Bioworld), MITF (STJ94134, St. John’s Laboratory), TYRP1 (ab235447, Abcam), DCT (NBP1-56058, Novusbio), PMEL17(#H1219, Santa Cruz and bs-17478R, Bioss) and MLANA (bs-0051R, Bioss and ET1610-47, HUABIO) were purchased as indicated.

#### 2.2.2 Cell culture and cytokine treatment

The melanin-rich human melanoma cell line MNT1 (22) was acquired from the China Center for Type Culture Collection (CCTCC). The cells were cultured in DMEM include 20% FBS (Meisen), 1% NEAA, 1% P/S, and different concentrations of IL-37, IL-19, CXCL1, CXCL2 or CXCL13 for 24 or 48 h at 37°C with 5% CO_2_.

#### 2.2.3 Human skin culture

Human skin samples were obtained from healthy adolescent foreskins after circumcision, following approval by donors and the Ethics Committee of the Third Xiangya Hospital of Central South University (Changsha, China. No.2022-S111). Briefly, subcutaneous tissue and fat were removed from the foreskin samples, and the latter were cut into 2 cm^2^ pieces. The skin pieces were placed on DMEM included with 10% FBS, 1%penicillin/streptomycin/amphotericin B and IL-37 (0, 50,100, 200 ng/ml) with the epidermis side up. The plates were incubated at 37°C with 5% CO_2_ for 5 days.

#### 2.2.4 Cell viability assay

Cell viability was evaluated using the CCK8 assay. Briefly, the MNT1 cells were plated in 96-well plates at the density of 2000-3000 cells/well, and treated with different concentrations of IL-37 for 24 or 48 h. Ten microliters CCK8 solution was added to each well and the cells were incubated for 2 h. The absorbance at 450 nm was measured using a microplate reader (PerkinElmer EnVision xcite, UK).

#### 2.2.5 RNA extraction and qRT-PCR

After being treated with cytokine for 24h, the cells were digested with trypsin and collected in 1.5 mL tubes, then the total cellular RNA was extracted using Fast total RNA extraction kit (FASTAGEN, Shanghai, China) and reverse-transcribed. Quantitative Real-time PCR (qRT-PCR) was performed using the qPCR SuperMix (TransGen) on Roche LightCycler480II (Basel, Switzerland). The relative gene expression levels were calculated by the comparative threshold cycle method (2^–ΔΔCT^). The primer sequences are shown in **Supplementary Materials** (**Table S1**).

#### 2.2.6 Fontana-Masson melanin staining

The cells stimulated with cytokine at different concentrations. After 24h, we immobilized the cells with 4% neutral paraformaldehyde for 20 min and washed with distilled water for 4-5 times, then 500μL Fontana ammonia-silver solution was added and the cells were incubated at 56°C for 30 min in the dark. The cells were rinsed with distilled water, incubated with 500μL hyposulphite for 5 min, and rinsed again with tap water for 1-2 min. Paraffin skin sections were stained using a Fontana-Masson Stain kit (G-ClONE, Beijing, China). Melanin granules were observed under an inverted microscope.

#### 2.2.7 Melanin quantification

The cells were treated with IL-37 for 48h. After collected, washed twice in PBS and counted, cells were resuspended in 1ml of 10% DMSO (Diluted with 1mM NaOH solution) and incubated at 80°C for 1h to dissolve the melanin. 200 μL of the mixture was moved to a 96-well plate and the absorbance value at 470 nm was measured using a multimode plate reader.

#### 2.2.8 Transmission electron microscopy (TEM)

After being treated with IL-37 for 48h, cells pellets were collected, and prefixed with 3% glutaraldehyde and refixed with 1% tetroxide. Then the pelleted were dehydrated with an acetone gradient (series of solutions from 30% to 100%), and permeated by a mixture of dehydrant and Epon812 embedding agent, following embedded with Epon812. Subsequently, the pelleted material was cut into thin (60-90nm) sections using a LEICA UC7rt ultramicrotome and collected onto copper mesh, then stained with uranyl acetate for 10-15 minutes and lead citrate for 1-2 minutes. The JEM-1400FLASH (LEOL, Japan) was used to observe the melanosomes.

#### 2.2.9 TYR activity measurements

The suitably treated cells were harvested, washed with PBS, counted, and incubated with 500μL 0.5% sodium deoxycholate solution at 4°C for 15 min, and thereafter at 37°C for 10 min to release TYR. After adding 1mL 0.1% L-DOPA substrate solution, 200μL aliquots were immediately dispensed in a 96-well plate. The absorbance at 475 nm was measured at 0 min (A0) and 30 min (A30) using a multimode plate reader. TYR activity was calculated as (A30-A0)/number of cells.

#### 2.2.10 Western blot analysis

After being treated with IL-37 for 48h, cells were lysed by RIPA lysis buffer (Thermo Fisher) supplemented with 1% Protease Inhibitor cocktail (Roche). Then the lysis mixture was further used for total protein extraction. Total protein concentration was measured using a BCA protein assay kit (KeyGEN Biotec). Equal amounts of protein per sample were separated using 10% SDS-PAGE (YEASON, Shanghai, China) and transferred onto a polyvinylidene fluoride (PVDF) membrane, then blocking with 5% milk. The membrane was incubated overnight with the primary antibody (1:1000 or 1:2000) at 4°C, followed by fluorochrome-conjugated secondary antibody (1:10000, LI-COR Biosciences) for 1h at 37°C. The fluorescence intensity of the bands was measured using an Odyssey CLx Imaging System (Li-COR Biosciences).

#### 2.2.11 Immunofluorescence (IF)

Cells treated with IL-37 for 24h were fixed with 4% neutral paraformaldehyde for 15 min, and permeabilized with 0.5% Triton X-100 for 10 min. After blocking with 5% BSA, the cells were incubated overnight with mouse anti-PMEL or rabbit anti-MLANA antibody (1:200 diluted with the blocking solution) at 4°C, followed by donkey anti-mouse or goat anti-rabbit Alexa Fluor 488 (Beyotime Biotech) secondary antibody (1:400) for 1h at 37°C. The cells were counterstained with DAPI for 5 min, and fluorescence intensity was evaluated using an inverted microscope.

#### 2.2.12 Statistical analysis

Data used R (4.0.3 version) and GraphPad (8.0.2 version) for processing and visualization. The two groups were compared by unpaired Student’s T test, and the multiple comparisons were performed by univariate analysis of variance (ANOVA). Nonparametric data was tested by Mann-Whitney U test. p <0.05 was considered statistically significant. (***p <0.001, **p <0.01, *p <0.05).

## 3 Results

### 3.1 Collection and evaluation of GEO datasets

**Figure 2:**
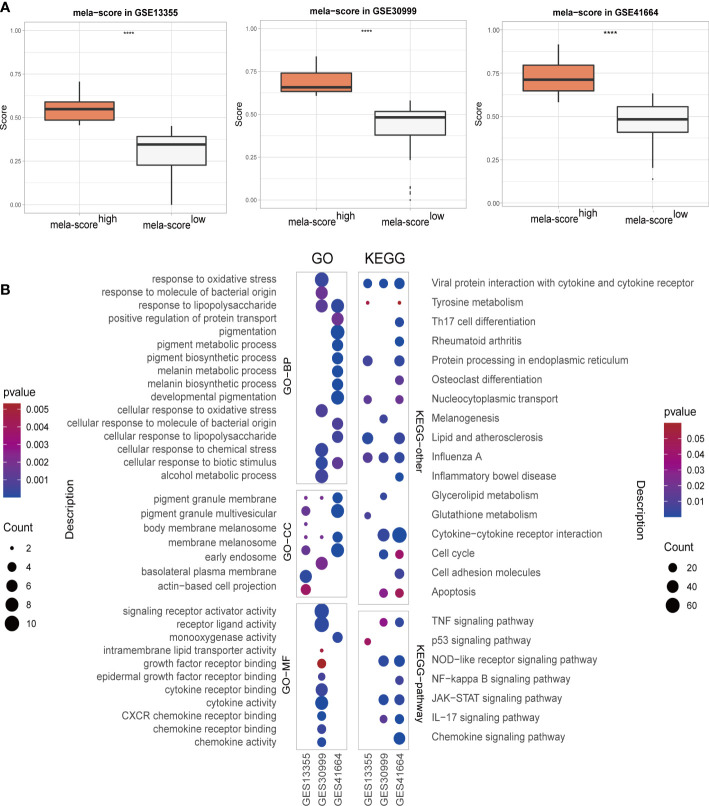
The overview of skin samples in 3 GEO datasets after regrouping. **(A)** Boxplot showing the weighted scores of melanogenesis-related genes. **(B)** GO and KEGG enrichment analyses results. ****P < 0.0001.

Transcriptomic GEO datasets of inflammatory diseases, such as psoriasis, atopic dermatitis (AD) and systemic lupus erythematosus (SLE), were preliminarily screened on the basis of sample type and size. Three psoriasis datasets (GSE13355, GSE30999 and GSE41664) were selected as the training set for screening DEGs. One dataset each of atopic dermatitis (GSE133477) and psoriasis (GSE117468) were used as validation sets (**Table 1**). The results of hclust and PCA showed that lesion samples were well distinguished from the healthy and non-lesion skin samples (**Figure S1**). Weighted score of melanogenesis-related genes (MITF, TYR, DCT, PMEL, MLANA, WNT4 and WNT2B) were calculated using ssGSEA (named mela-score), and the lesion samples were divided into low and high melanogenesis groups according to the median score. As shown in the boxplots in **Figure 2A**, the mela-scores of the high and low groups were significantly different, suggesting that our grouping was feasible. The DEGs between the two groups were screened from 3 datasets, and the top 200 DEGs (ordered by P-value) were used in GO and KEGG enrichment analyses. Functional annotation indicated that the DEGs were most likely related to pigmentation and skin inflammation (**Figure 2B**, **Figures S2**, **S3**).

### 3.2 Evaluation of inflammatory genes

**Figure 3:**
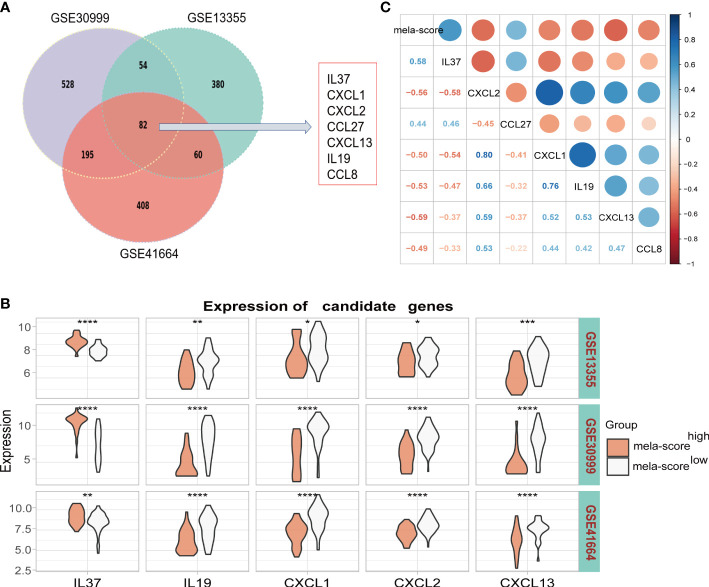
Identification of candidate genes. **(A)** Seven inflammatory genes were screened from the overlapping DEGs of 3 datasets. **(B)** The mRNA expression levels of 7 candidate genes in the datasets. **(C)** The correlation between candidate genes and mela-score. *P < 0.05, **P < 0.01, ***P < 0.001,****P < 0.0001.

There were 82 genes in the top 5% DEGs that were common to all three datasets of the training set, and included 7 inflammation-related genes (**Figure 3A**). IL-37 and CCL27 were highly expressed in the mela-score ^high^ group, while CXCL1, CXCL2, CXCL13, IL-19 and CCL8 were expressed at low levels (**Figure 3B**). In addition, all seven genes were highly correlated with the mela-score (**Figure 3C**), and therefore used to construct prediction models.

### 3.3 Establishment of predictive models

**Figure 4:**
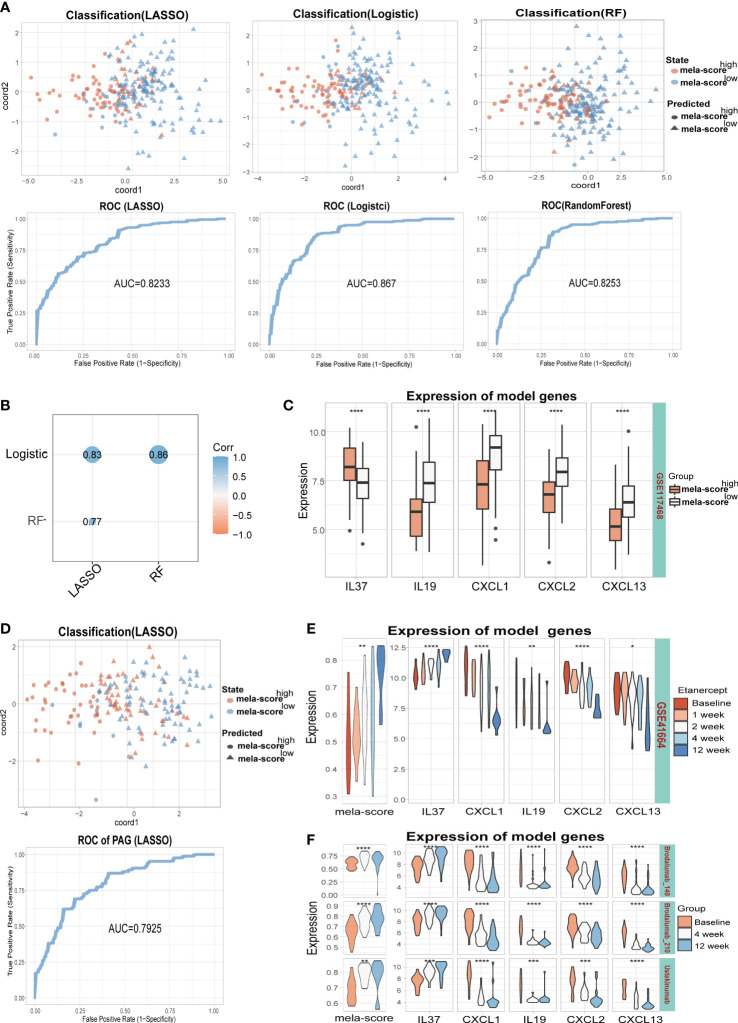
Training and testing models were built by different algorithms (LASSO, Logistic and RF). **(A)** The classification ability of the 3 models on training datasets (GSE41664, GSE30999, GSE13355). **(B)** Correlation between model scores in training data. **(C)** The expression levels of 5 model genes. **(D)** The classification ability and ROC of the LASSO model in testing dataset (GSE117468). The mela-score and expression levels of 5 inflammatory genes in GSE41664 **(E)** and GSE117468 **(F)**. *P < 0.05, **P < 0.01, ***P < 0.001,****P < 0.0001.

In order to identify the core inflammatory genes involved in aberrant pigmentation and assess their predictive value for inflammation-induced skin pigmentation, we constructed predictive models using LASSO, logistic regression and RF algorithms. IL-37, CXCL1, IL-19 and CXCL2 were used for the LASSO model after reducing the number of genes by penalty regression to optimize model performance (**Figures S4A, B**). Similarly, IL-37, CXCL13 and IL-19 were screened out by stepwise regression in the logistic regression model (ordered by coefficients of stepAIC). In addition, IL-37, CXCL13 and IL-19 were screened out by adjusting the mtry and ntree parameters in RF model (**Figures S4C, D**). Thus, IL-37, IL-19 and CXCL13 were used in all three models. As shown in **Figure 4A**, all models were able to classify samples into the high and low mela-score groups with high accuracy, since the area under the ROC curve (AUC) was greater than 0.8. Furthermore, the predictive outcomes of the three model genes showed significant positive correlation (**Figure 4B**), indicating their potential as indicators for skin pigmentation outcomes after inflammation.

In the testing set, all model genes except IL-19 were differentially expressed between the high and low melanogenesis groups (**Figure 4C**, **Figure S5A**). Furthermore, the samples were well classified by the 3 models (AUC>0.70, **Figure 4D**, **Figure S4E**, **Figure S5B**), and the predictive outcomes were positively correlated (**Figure S4F**, **Figure S5C**). Some of the patients in GSE41664 and the testing datasets (GSE117468 and GSE133477) underwent biological therapy, the mela-scores and expression levels of the 5 inflammatory genes in skin lesions were affected by the treatments. In GSE41664, mela-score and IL-37 expression levels were gradually up-regulated after etanercept treatment, while CXCL1, IL-19, CXCL2 and CXCL13 were down-regulated (**Figure 4E**). Similar trends were observed for the mela-score and expression levels of 5 inflammatory genes after Brodalumab (140/210 mg) and Ustekinumab treatment in the GSE117468 dataset (**Figure 4F**). In the GSE133477 dataset, mela-score and IL-37 expression levels were also up-regulated with Crisaborole treatment, while CXCL1 and CXCL2 were down-regulated, and IL-19 and CXCL13 were unaltered (**Figure S5D**, complete results in the **Supplementary Materials**). Our findings suggest that IL-37, CXCL13, CXCL1, CXCL2 and IL-19 may be involved in melanogenesis regulation, and 3 models based on these genes can predict the pigmentation outcomes of post-inflammation skin with relatively high accuracy.

### 3.4 IL-37 promotes melanogenesis in MNT1 cells and human skin

**Figure 5:**
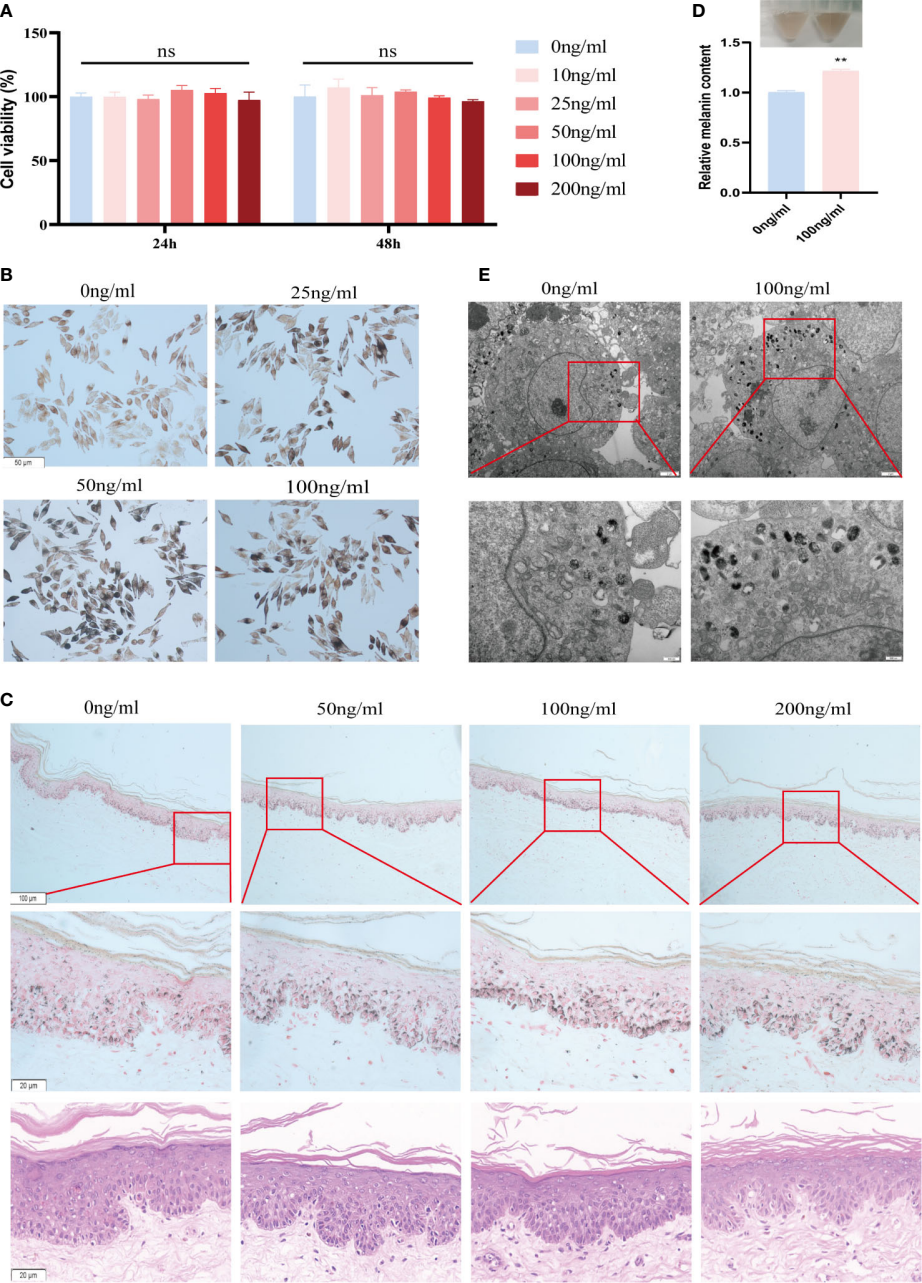
IL-37 increases melanin content in MNT1 cells and human foreskin. **(A)** Viability of MNT1 cells. **(B, C)** Representative images of Fontana-Masson-stained MNT1 cells **(B)** and human foreskin **(C)** showing melanin granules. **(D)** Melanin content was measured by NaOH method. **(E)** Melanosomes was observed by TEM. **P < 0.01. ns, No significant difference.

Among the 5 model inflammatory genes involved in aberrant skin pigmentation, IL-37 was the only factor that was positively correlated with mela-score and affected by biologics treatment. Therefore, we then focus on verified the role IL-37 in the MNT1. As shown in **Figure 5A**, IL-37 had no significant effect on cell viability at concentrations below 200ng/ml. Furthermore, different concentrations of IL-37 (25, 50 and 100 ng/mL) increased melanin content in MNT1 cells following 24h culture (**Figure 5B**, **Figure S6A**), while CXCL1, CXCL2, CXCL13 and IL-19 decreased melanin content (**Figure S7A**). Human foreskin pieces cultured in the presence of IL-37 also showed increased melanin density without significant cell damage or inflammation (**Figure 5C**, **Figure S6B**). Meanwhile, we further detected melanin content by the NaOH method, and observed the melanosomes by TEM after IL-37 treatment. The results showed that the melanin content (**Figure 5D**), and the number of melanosomes in stage III-IV (**Figure 5E**) were increased after IL-37 treatment.

### 3.5 IL-37 upregulates melanogenesis-related genes

**Figure 6:**
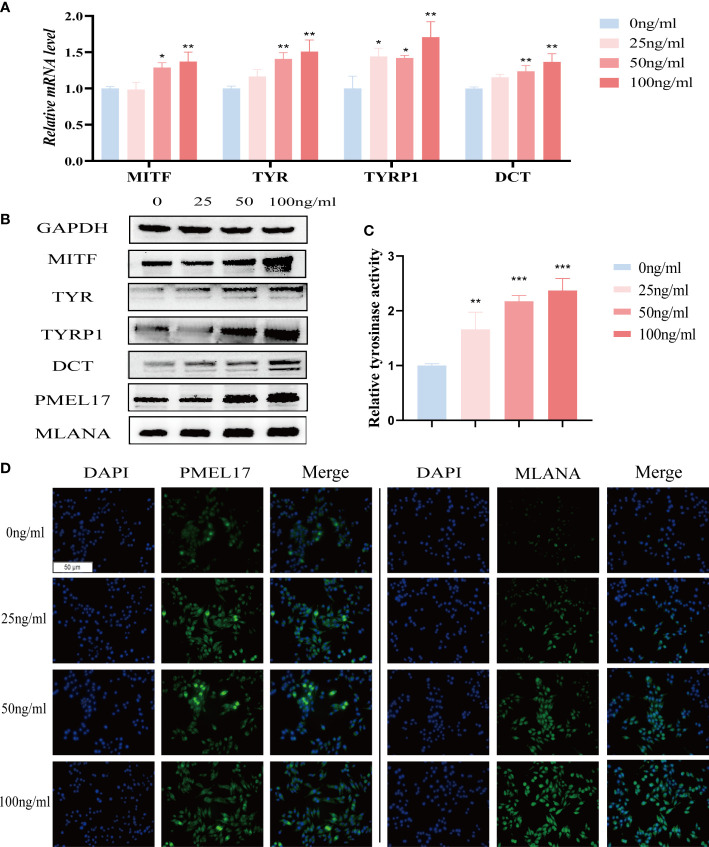
The role of IL-37 in melanogenesis. MNT1 cells were treated with different concentrations of IL-37. **(A)** qRT-PCR was used to examine the mRNA levels of melanogenesis-related genes. **(B)**The protein levels of MITF, TYR, TYRP1, DCT, PMEL17 and MLANA were analyzed by Western blotting. **(C)** Tyrosinase activity was measured by L-DOPA assay. **(D)** representative immunofluorescence images showing the distribution of melanosomes. (N = 3, ANOVA, error bar represents mean ± SEM, *P < 0.05, **P < 0.01, ***P < 0.001).

We further investigated the effect of IL-37 on melanogenesis-related genes, including MITF, TYR, TYRP1 and DCT. IL-37 significantly upregulated the expression of above genes in MNT1 cells (**Figures 6A, B**). Meanwhile, IL-37 also promoted the tyrosinase activity in MNT1 cells in a concentration-dependent manner (**Figure 6C**), and increased the levels of melanosomes markers PMEL17 and MLANA (**Figures 6B, D**). The effects of CXCL1, CXCL2, CXCL13 and IL-19 on pigment cells were also examined. After being treated with CXCL1, CXCL2, CXCL13 or IL-19 for 24h respectively, melanogenesis-related genes were down-regulated at different level in MNT1 cells (**Figure S7B**).

## 4 Discussion

The aim of this study was to identify the core inflammatory factors involved in melanogenesis regulation, and the predictive markers of the outcome of post-inflammatory skin pigmentation. We found that melanogenesis-related DEGs were primarily involved in inflammation, which is consistent with the aberrant pigmentation frequently observed in inflamed skin lesions. We constructed LASSO, Logistic and RF predictive models using five core inflammatory genes including IL-37, CXCL13, CXCL1, CXCL2 and IL-19, which could accurately classify between the high and low pigmented samples. Furthermore, IL-37 showed a strong positive correlation with melanogenesis.

Aberrant pigmentation is very common in inflammatory skin diseases. However, since multiple factors regulate inflammation-induced pigmentation, it is challenging to accurately predict that the occurrence of post-inflammatory pigmentation outcomes. Using the existing omics data, we screened out inflammatory factors such as IL-37, CXCL13, CXCL1, CXCL2 and IL-19 that may be involved in the regulation of post-inflammatory pigmentation, and found that only IL-37 was positively correlated with melanogenesis, while the rest were negatively correlated. These results suggest that inflammation acts as a two-edged sword in melanogenesis. Although our established models showed good predictive performance and high accuracy, we did not include all kinds of inflammatory skin diseases in the model. Given the lack of reliable reference data on skin pigmentation, our model will have to be verified on more clinical samples.

IL-37 is a member of the IL-1 family that can suppress immune and inflammation responses by inhibiting transcription of pro-inflammatory genes (23, 24). It plays a regulatory role in inflammatory diseases such as psoriasis (25), AD (26) and lupus (27). The IL-37 localized on granular layer that is frequently absent in psoriasis, resulting decreased expression of IL-37 in lesion skin (28). IL-19, a member of the IL-10 family, has been reported to be highly expressed in AD and psoriasis, may be important for linking Th17 with Th2 (29, 30). Keratinocytes produce large amounts of chemokines, such as CXCL1, CXCL2. CXCL1 and CXCL2 recruit neutrophils, causing a breach of epidermal hyperproliferation, intraepidermal neutrophilic microabscess, which are the typical features of psoriasis (31). Similarly, CXCL13 expression was reported to be positively correlated with the severity of psoriasis (32). The relationship between these five inflammatory factors and psoriasis has been reported, but whether they are related to hyper-pigmentation and hypo-pigmentation is unknown. Although the role of these genes in melanogenesis has not been reported so far, there are still some hints. For example, it has been reported that IL-37, IL-19, CXCL1, CXCL2, and CXCL13 could regulate NF-κB, MAPK, PI3K/AKT, STAT3 or other signaling pathways (28, 33–37), which play important roles in melanogenesis (38, 39). In our study, we found that IL-37 upregulated melanogenesis-related genes such as MITF, TYR, TYRP1 and DCT in MNT1 cells, enhanced tyrosinase activity and increased melanin content. In addition, CXCL13, CXCL1, CXCL2 and IL-19 could down-regulated several melanogenesis-related genes at the mRNA levels. However, whether those inflammatory factors regulate melanogenesis through the signaling pathways mentioned above remains to be further verified experimentally.

According to our analysis, with the treatment of immunosuppressants in psoriasis, the expression of IL-37 increased gradually, while IL-19, CXCL1, CXCL2, CXCL13 decrease gradually. Psoriatic lesions are typically hypo-pigmented during active inflammation, while resolution of the inflammatory process increases the risk of hyper-pigmentation. Therefore, combining the existing reports and our findings, here we present a conjecture: during the active inflammation of psoriasis, due to the decreased expression of IL-37, which is positively associated with melanogenesis, and the increased expression of IL-19, CXCL1, CXCL2, and CXCL13, which are negatively associated with melanogenesis, hypo-pigmentation in psoriatic lesions emerges. When the inflammatory response subsides with treatment, the expression of IL-37 is gradually upregulated, while IL-19, CXCL1, CXCL2, CXCL13 are gradually down-regulated. If the levels of these inflammatory factors do not return to a balanced state, with excessive expression of IL-37 and low expression of IL-19, CXCL1, CXCL2 and CXCL13, the hyper-pigmentation in psoriatic lesions may occur. Therefore, we may predict the possible outcomes of post-inflammatory pigmentation abnormalities basing on our evaluation models by measuring the expression levels of IL-37, IL-19, CXCL1, CXCL2 and CXCL13. In future work, more clinical samples and *in vitro* and *in vivo* experiments are needed for hypothesis validation.

There are several limitations in our study that ought to be considered. First, very few transcriptomic data sets of inflammatory skin diseases are available at present, and are limited in terms of disease, sample size and sample types. Second, it is difficult to collect skin tissue samples with post-inflammatory hyper- or hypo-pigmentation for pre-model screening and post-clinical sample validation. Third, specific measures to prevent or treat post-inflammatory pigmentation abnormalities need to be further explored.

In conclusion, IL-37, CXCL1, CXCL2, CXCL13 and IL-19 are involved in regulating melanogenesis and could be novel indicators in evaluation models for predicting outcomes of post-inflammatory pigmentation abnormalities basing on machine learning. Our study has shown that machine learning is an effective method to systematically evaluate genes involved in skin pigmentation.

## Data availability statement

The datasets presented in this study can be found in online repositories. The names of the repository/repositories and accession number(s) can be found in the article/**Supplementary Material**.

## Ethics statement

The studies involving human participants were reviewed and approved by the Ethics Committee of the Third Xiangya Hospital of Central South University. The patients/participants provided their written informed consent to participate in this study.

## Author contributions

QZ and YZ conceptualized and designed this study. YZ conducted the experiments. HZ, LJ, CF, and LZha collected the data. YH and YZ analyzed the relevant data. FZ, XZ, and LZhu complete data visualization. YZ wrote the manuscript. JC, QZ, and JH provided funding support. JC and QZ reviewed and corrected the manuscript. All authors read and approved the final manuscript.

## Funding

This work was supported by the National Natural Science Foundation of China (No. 82073421, No. 82103704); the Natural Science Foundation of Hunan Province (No. 2021JJ20089, No. 2021JJ40924); the Wisdom Accumulation and Talent Cultivation Project of the Third Xiangya Hospital of Central South University (No. YX202007); The science and technology innovation Program of Hunan Province (No. 2021RC3035); the Fundamental Research Funds for the Central Universities of Central South University (No. 2022ZZTS0961).

## Acknowledgments

We thank the generous contributors of 5 GEO data sets (GSE13355, GSE30999, GSE41664, GSE117468, and GSE13355), these data sets provided the necessary basis for our analysis.

## Conflict of interest

The authors declare that the research was conducted in the absence of any commercial or financial relationships that could be construed as a potential conflict of interest.

## Publisher’s note

All claims expressed in this article are solely those of the authors and do not necessarily represent those of their affiliated organizations, or those of the publisher, the editors and the reviewers. Any product that may be evaluated in this article, or claim that may be made by its manufacturer, is not guaranteed or endorsed by the publisher.
