# Supplementary material for: Establishment and validation of evaluation models for post-inflammatory pigmentation abnormalities
Source: Front Immunol. 2022 Oct 27;13:991594. doi: 10.3389/fimmu.2022.991594 (PMC9646533; doi:10.3389/fimmu.2022.991594)
Supplement: Supplementary file 1 [file DataSheet_1.pdf]

Supplementary Material

1 Supplementary Figures and Tables

1.1 Supplementary Tables

Supplementary Table 1. Information of primer sequences:

Gene	Forward	Reverse
GAPDH	CTCTGCTCCTCCTGTTCGAC	GCCCAATACGACCAAATCC
MITF	AAATACGTTGCCTGTCTCGG	TGTTGGGAAGGTTGGCTGGA
TYR	TCAGCCCAGCATCATTCTTC	GGCATCCGCTATCCCAGTAA
TYRP1	ACCAGAGGGTTCTCATAGTCAG	TTCTCAAATTGTGGCGTGTT
DCT	GGGCAGCGAGACCAGACGAT	TTGGCAATTTCATGCTGTTTCTTC

1.2 Supplementary Figures

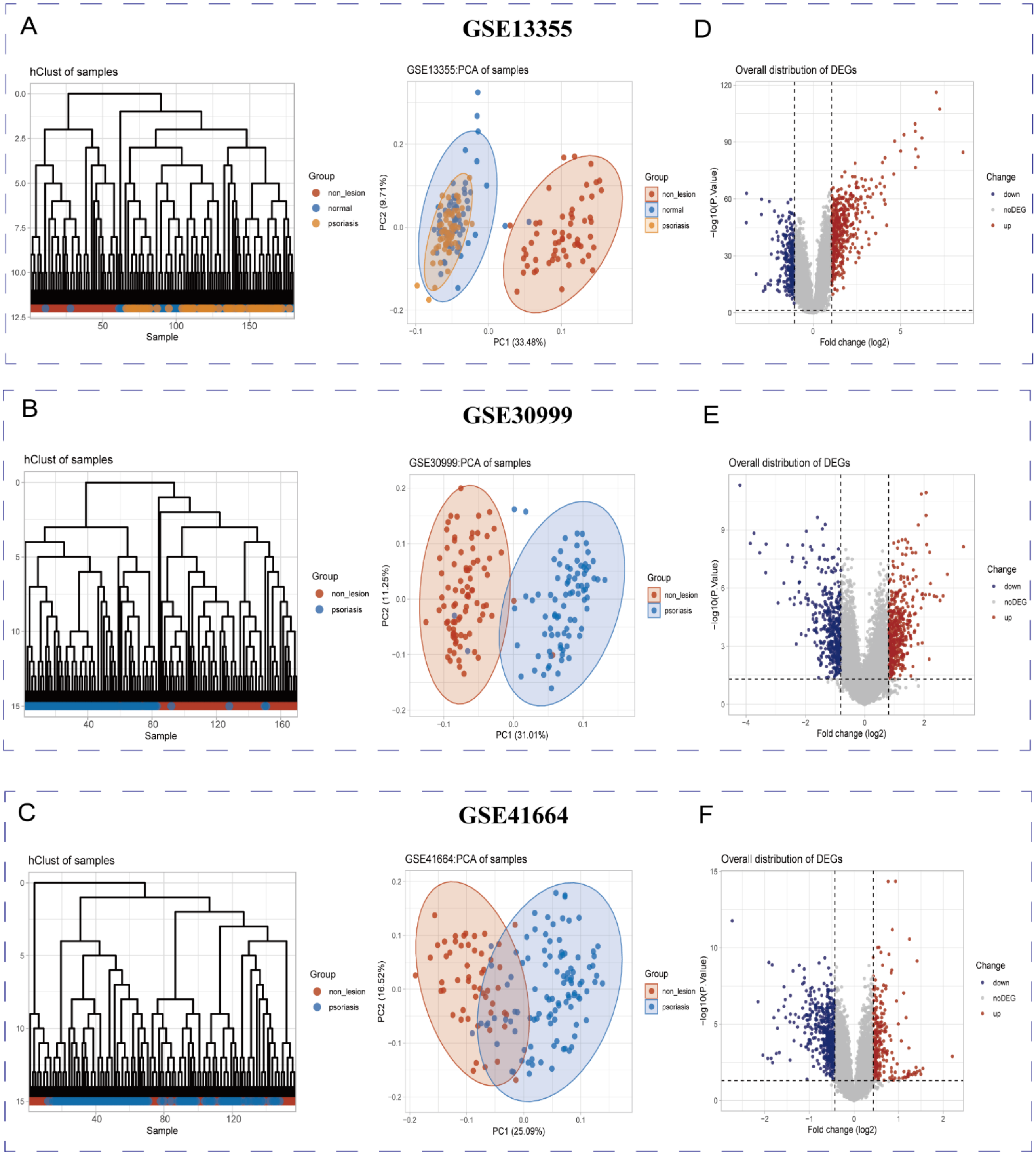

Supplementary Figure 1. Overview of the 3 datasets. (A-C) The Hierarchical Clustering (hclust) and Principal Component Analysis (PCA) of three datasets. (D-F) Volcano plot was used to show the distribution of DEGs after regrouping according to the mela-score.

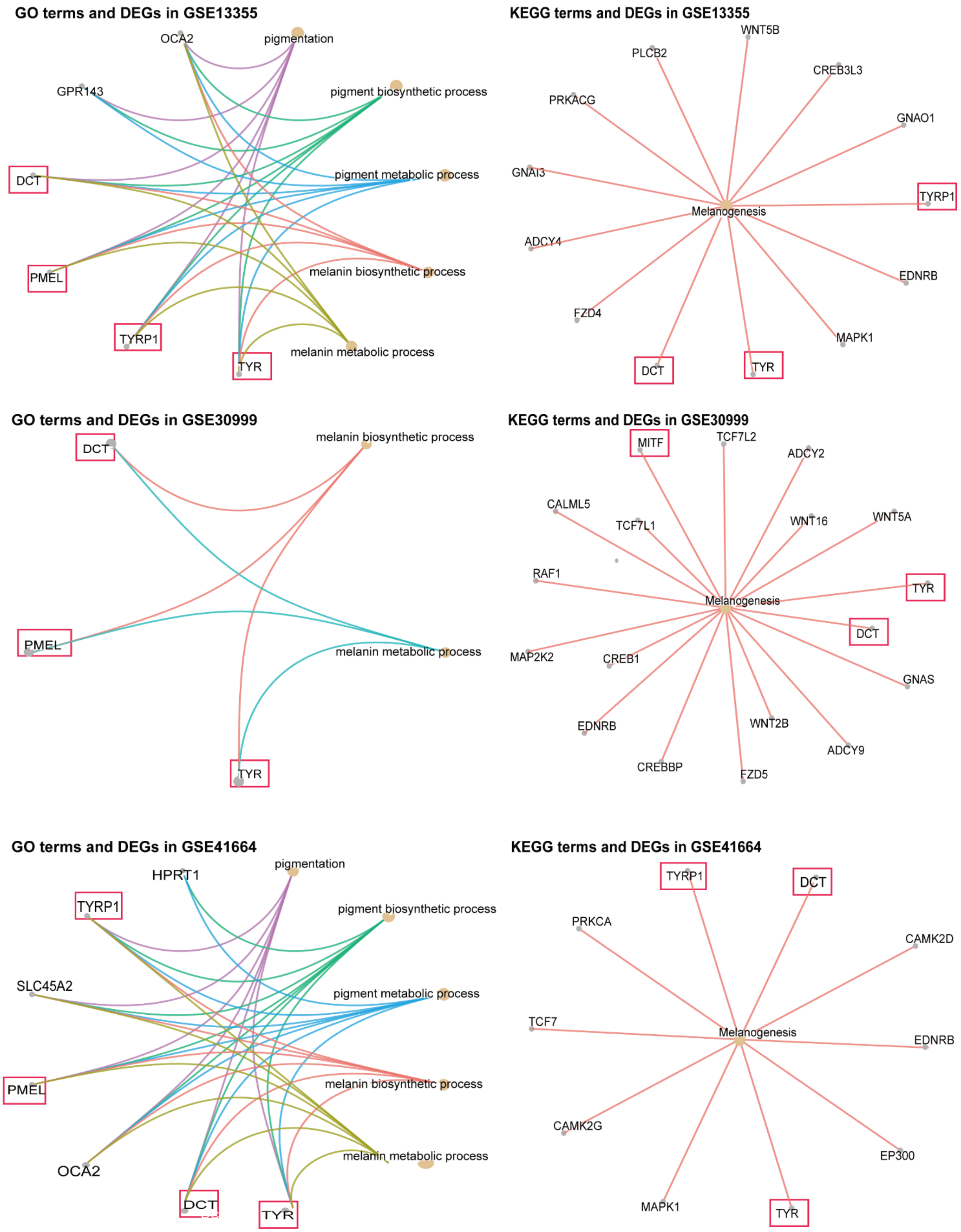

Supplementary Figure 2. The genes included in the pigmentation-related entries in the GO/KEGG enrichment analysis.

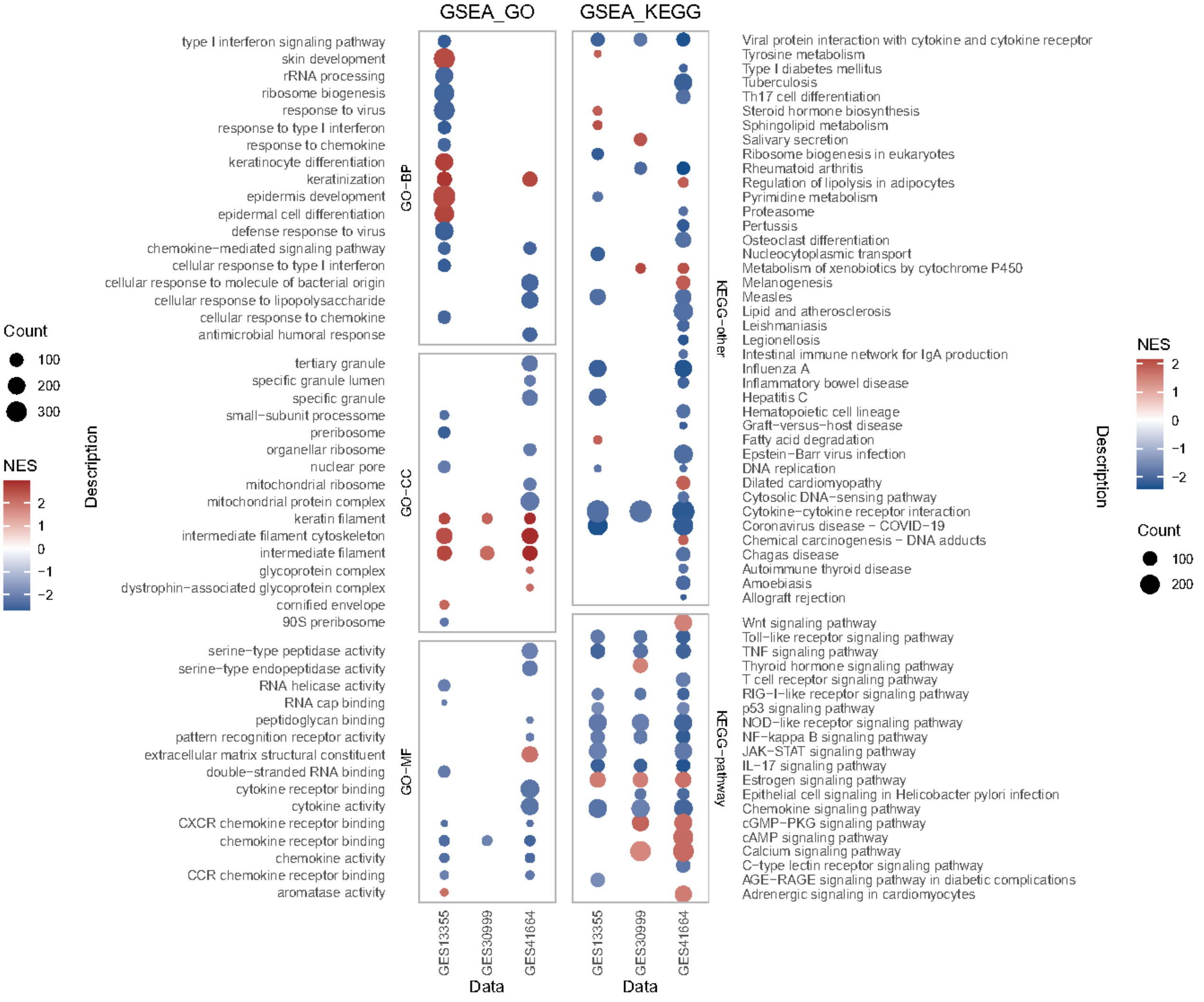

Supplementary Figure 3. GSEA enrichment results.

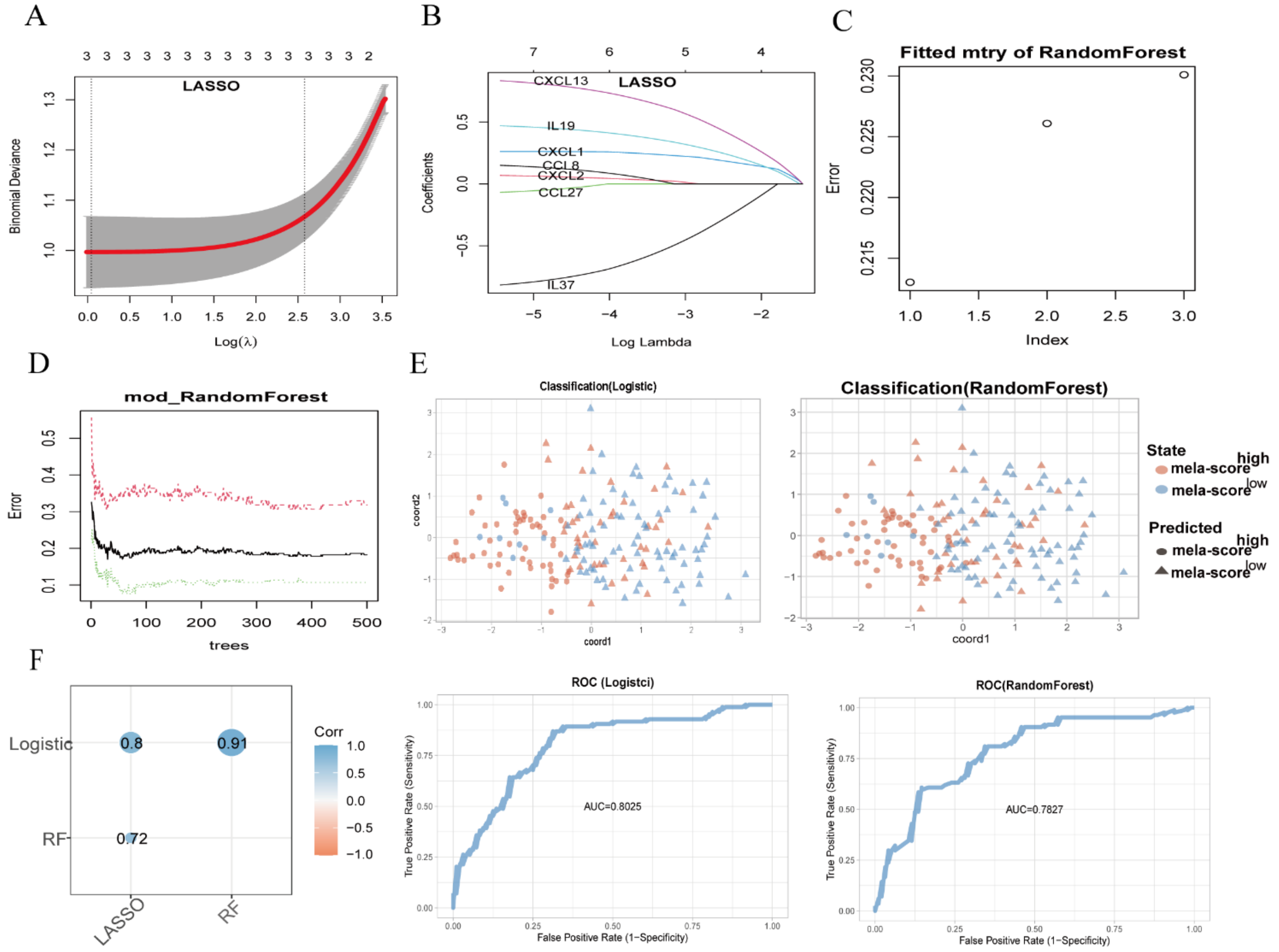

Supplementary Figure 4. Training and testing models that established by different methods. (A-B) LASSO through penalized regression screened out inflammation genes; the mtry(C) and ntree (D) parameters were adjusted in Random Forest (RF) models. (E) the classification performance and ROC of models in GSE117468. (F) the Pearson correlation coefficients between model scores in GSE117468.

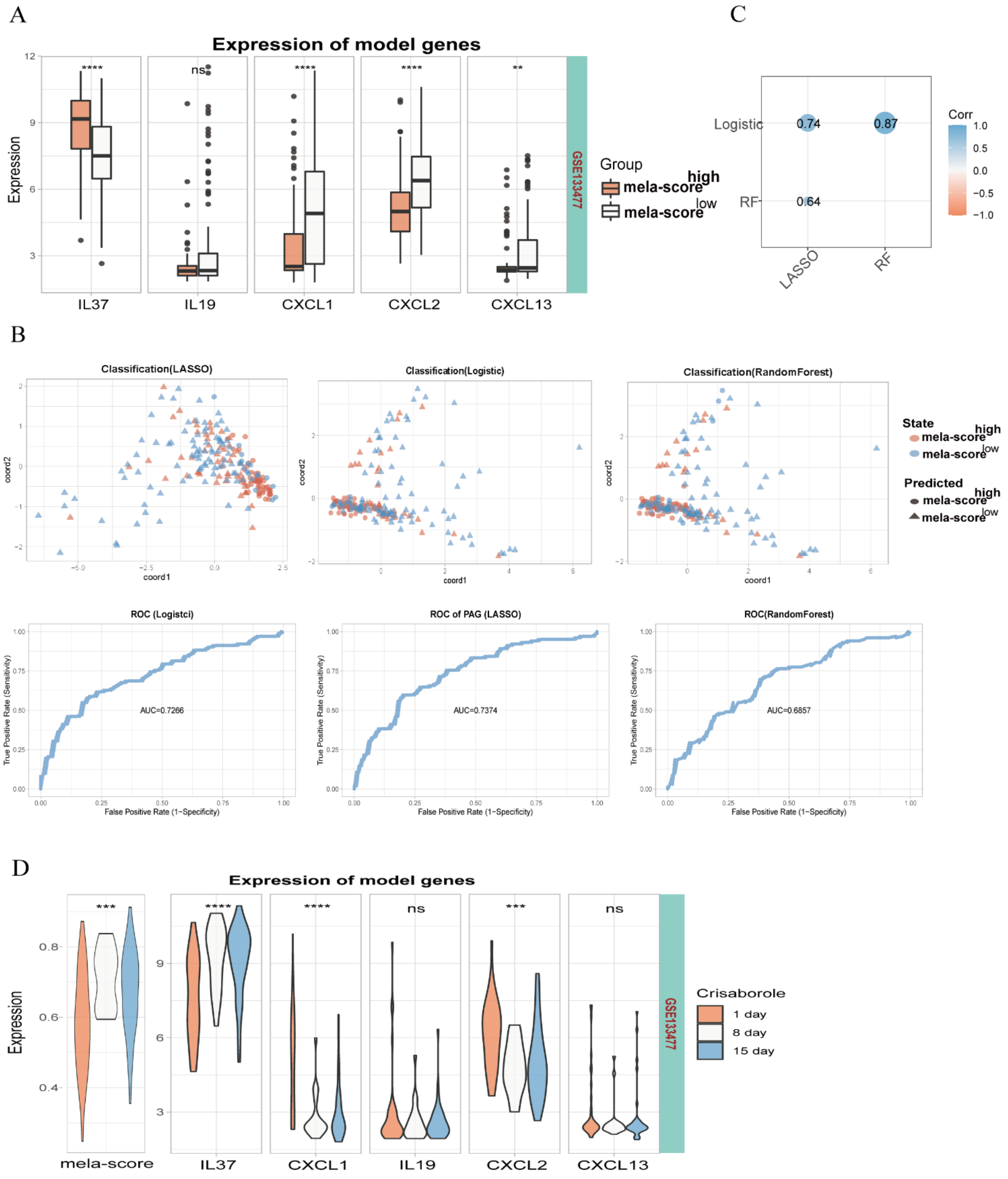

Supplementary Figure 5. Performance of models in GSE133477. (A) the expression of 5 model genes. (B) the classification performance of 3 models. (C) the Pearson correlation coefficients between model scores. (D) The mela-score and 5 inflammation genes expression grouped by treating time.

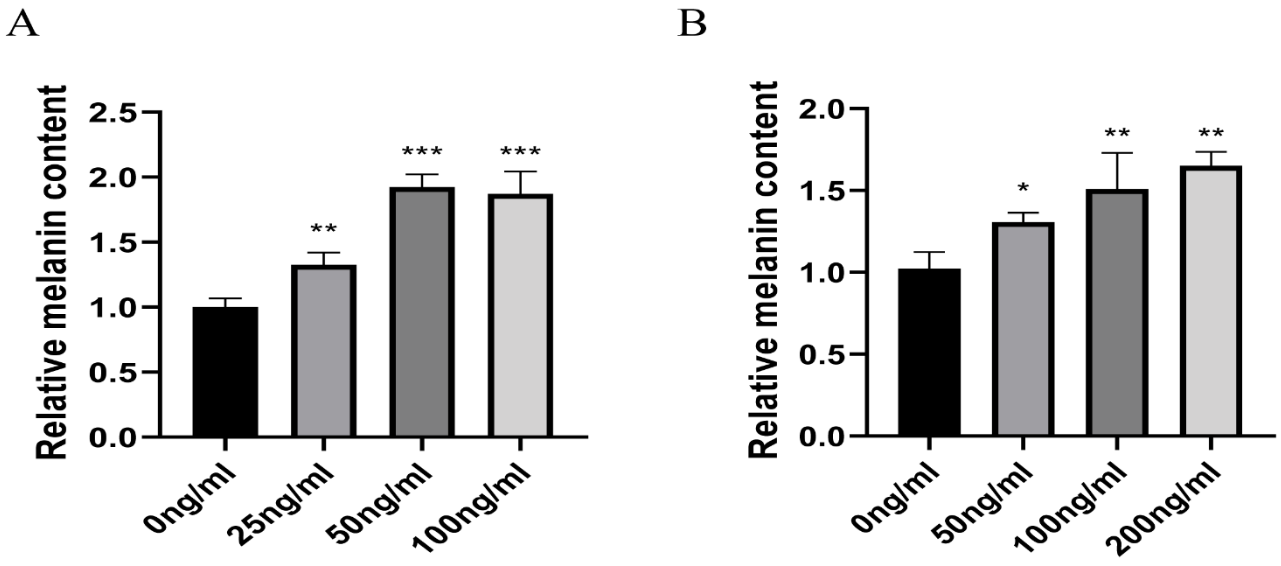

Supplementary Figure 6. IL-37 increases melanin content in MNT1 cells and human foreskin. Melanin granules shown in representative images of Fontana-Masson stained MNT1 cells (A) and human foreskin (B) were quantified by ImageJ. ($N \geqslant 3$, ANOVA, error bar represents mean $\pm$ SEM, *$P < 0.05$, **$P < 0.01$, ***$P < 0.001$)

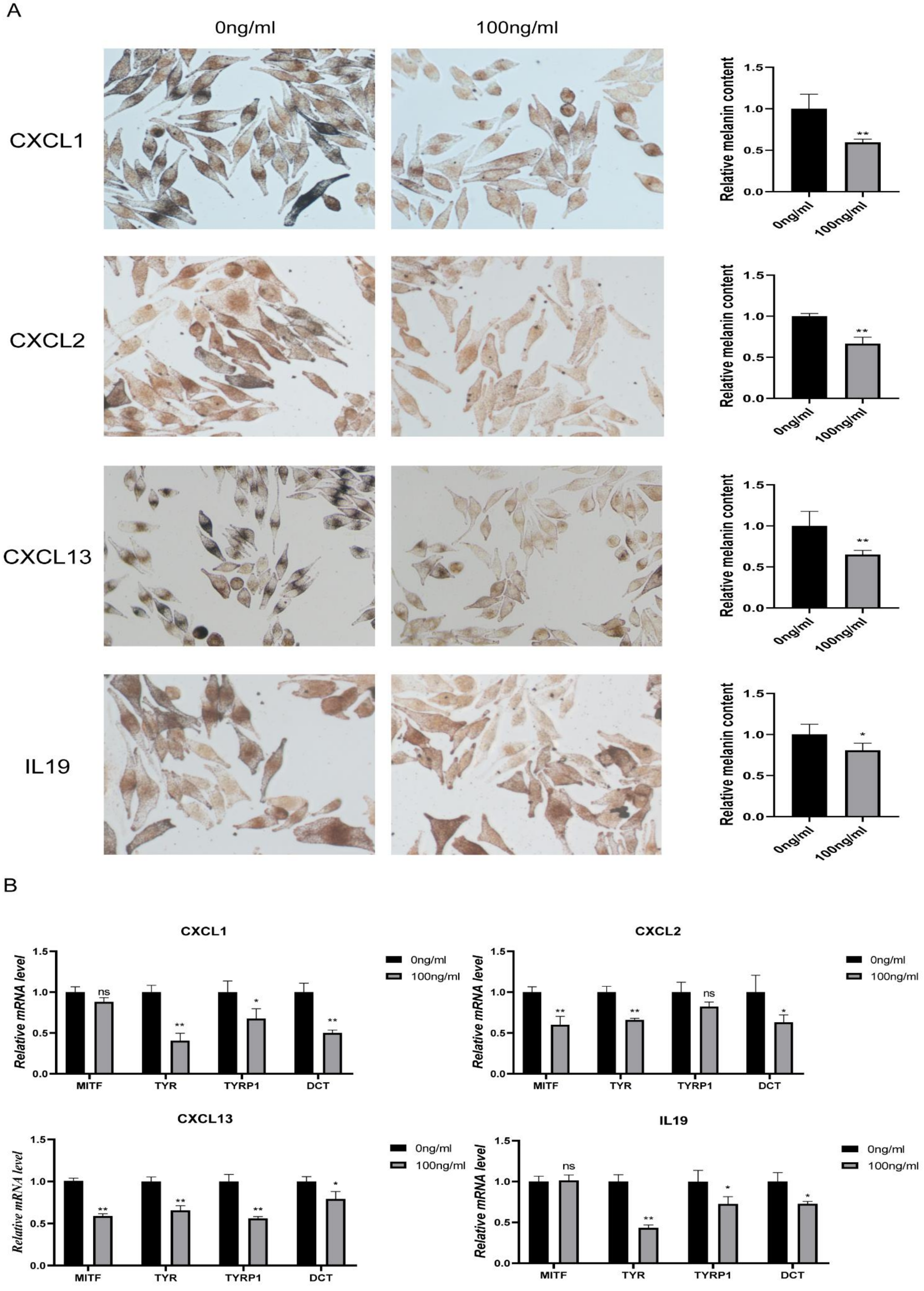
A
0ng/ml
100ng/ml
CXCL1
CXCL2
CXCL13
IL19
Relative melanin content
1.5
1.0
0.5
0.0
0ng/ml
100ng/ml
**
*
B
CXCL1
CXCL2
CXCL13
IL19
Relative mRNA level
MITF
TYR
TYRP1
DCT
ns
0ng/ml
100ng/ml

Supplementary Figure 7. The role of inflammatory factors in melanogenesis. MNT1 cells were treated with CXCL1, CXCL2, CXCL13 or IL-19 for 24h. (A) Representative images of Fontana-Masson-stained showing melanin granules, and melanin granules were quantified by ImageJ. Scale bar, 20μm. (B)The mRNA levels of melanogenesis-related genes. (N≥ 3, Unpaired Student's t test, error bar represents mean ± SEM, *P < 0.05, **P < 0.01)
